# Sustainable and green manufacturing of gravure printing cylinder for flexible packaging printing application

**DOI:** 10.1038/s41598-022-15893-1

**Published:** 2022-09-28

**Authors:** Bhavna Sharma, Sauraj Singh, Arun Pandey, Dharm Dutt, Anurag Kulshreshtha

**Affiliations:** 1 Department of Paper Technology, Indian Institute of Technology Roorkee, Saharanpur Campus, Saharanpur, 247001 India; 2 Department of Materials Engineering, Indian Institute of Science, Bangalore, 560012 India; 3 Afflatus Gravures Private Limited, Sector 68, Noida, 201307 India

**Keywords:** Environmental sciences, Energy science and technology, Engineering

## Abstract

Rotogravure printing cylinders are engraved by electro-mechanical engraving (EME) process in India used for printing purpose. But this process has drawbacks of the emissions of hazardous gases, solid and water pollution. EME cylinders are better in cell size, depth and needed higher copper and chrome plating thickness. By laser engraving (LE) copper and chromium thickness were reduced by 75 µm and 5 µm in a cylinder by laser engraving with also a reduction in power consumption and plating time. The carbon footprints were also reduced by 227 g per cylinder with a cost-effective solution for rotogravure printing process.

## Introduction

**Figure 1:**
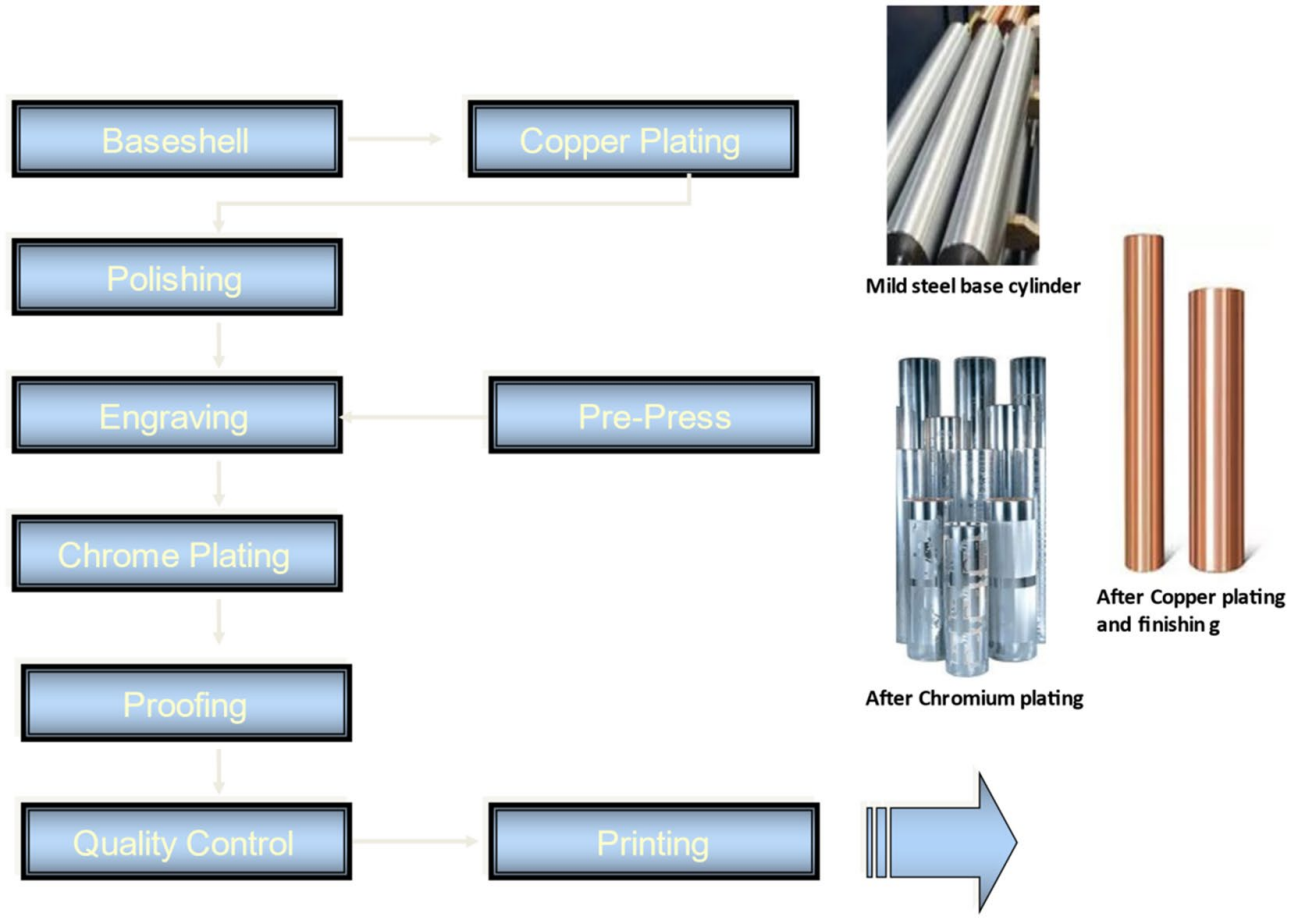
Flow chart showing the EME cylinders making processes.

The consumption trend of the flexible packaging market rapidly growing globally, and it was anticipated to grow from USD 102 billion in 2017 and to reach USD 132 billion by 2022, at an expected compound annual growth rate (CAGR) of 5.2%. Gravure printing also named as rotogravure printing or Intaglio printing was principally long-run or higher production quantity, high-speed, and high-quality printing method^1^. Printing is the key process of packaging to attract the consumer and promote the sales appeal of a package. Printing was defined by any reproduction activity of text and/or images in which, with the use of an image carrier like printing cylinder or plate for transfer of ink on paper or polymer substrate^2^. For printing the flexible packaging materials following are required for the printing process like printing cylinder, inks, solvents, substrate and printing machine mainly. Printing cylinder is a copper-plated and polished surface like a mirror-image appearance without scratches and lines. This cylinder was used for digging the engraving cell on the surface of copper plated cylinder to hold the ink so that only the intentional lines would be able to transfer the ink. The engraving process could be done by mechanical, chemical, laser and electro-chemical or electro-mechanical method^3–6^ The chemical etching method of engraving was based on using strong acid or mordant (e.g. ferric chloride) to remove the copper metal from a designated area of the unprotected parts of a metal surface to create a design in intaglio (gravure cylinder) on the plated and polished copper metal as per required graphic design. The chemical etching method was the manual method and variable factors like strength of chemical, etching perfection were having the drawback of cell variation so later on, this process was replaced by EME^7^. EME was the process, where a cell was engraved on the hard copper plated cylinder by a fully automatic process^8^. The cylinder cell depth was around 20–50 µm and had highly précised and consistent engraved cells^9^. Electromechanical engraving has worked successfully for decades with organic solvent-based inks. Fig. 1 shows the flow chart of the cylinder making by EME processes. Some major issues associated with cylinder making process like high emission of VOCs in the printing process, electroplating process of copper, nickel and chrome-plating and cost of effluent treatment along with adherence with strict legal compliances^10–12^. Therefore, efforts had been made to use an environmentally benign approach to reduce the VOCs emission and materials consumption in-cylinder making process for flexible packaging^13^.

Solvent-based ink did not pose a drying problem due to its highly volatile nature with an electromechanical engraving system. The existing electromechanical engraving system was found unsuitable for water-based ink due to the transfer of high ink volume on printing substrate which created drying issues within the specified travel time of printing film from one station to the next printing station^14^. The laser-induced engraving was the latest cutting-edge technological development in engraving techniques. In which the depth of the cell was drastically reduced for achieving the same printing characteristics compared to electronic engraving^9^. Depth of the cell reduces in LE due to non-contact digging of engraving cells by laser. The laser technique is very useful for engraving cells very precisely and accurately on coated copper.

Several studies were conducted to reduce VOCs emissions in the printing process but none of them was focused on the technological development of gravure cylinders and reduction in carbon footprints in the production of printing cylinders. Only a few studies focused on engraving methods and advancements in cylinder-making systems were available.

In India, National Green Tribunal (NGT) and Central Pollution Control Board (CPCB) forced the printing industries to reduce hazardous gases especially during chrome and copper-plating operations^15^. Most of the chrome-plating industries either shut down or shifted to remote locations in many parts of India^16^.

Laser engraving is a highly advanced technology in the field of rotogravure printing cylinder manufacturing. LASER (light amplification by stimulated emission of radiation) is consisting of three basic steps i.e., absorption, spontaneous emission, and stimulated emission. When engraving was performed by stylus, control over the depth and width is depended on the actual condition of the stylus. Class IV laser is used for this engraving process. Lasers deliver the ability to very precise and accurately deliver huge amounts of energy into confined regions of a material or surfaces in order to achieve an anticipated response. The absorption coefficient, which is derived from the material’s dielectric function and conductivity, regulates the absorption of light as a function of depth but these mechanisms for absorption strongly depend on the type of material. In laser engraving process, vaporizes materials into fumes takes place in the process. The laser beam acts as a carve, cutting of copper by eliminating copper plating layer from the surface of the printing cylinder. Vaporization temperature of copper is approximately 2595 °C. The laser beam hits the polished copper plated cylinder surface with enormous levels of energy to generate the high heat required for vaporization of copper plating. In laser etching process for engraving, laser beams melt the copper plated surface to change its roughness; laser engraving sublimates the material surface to create deep crevices (engraving cells) within milliseconds. The effects formed by this laser energy interacting with targeted material depend upon the wavelength, power level of the laser, absorption characteristics and chemical composition of the material^17^. By this process, copper surface instantly absorbs enough energy to change from solid to gas without ever becoming a liquid phase in whole mechanism. It is noteworthy here that laser engraving process area must having the facility of fume extraction system to keep the work environment safe and an air knife to protect the laser’s lens also.

Laser engraving on cylinders is highly precise and accurate compared to electromechanical engraving^18^. Laser engraving technology did not comprise the use of any kind of tool bits and diamond stylus, which contacted or dig the polished copper surface for engraving and possibly will wear out. To provide resistance to wear during long printing runs, the engraving cells or images on cylinder were protected. The coating layer of chromium was electroplated onto the cylinder’s surface within 10–12 µm for electromechanical engraving and 6–8 µm for the laser engraving cylinder. Therefore, it made it possible to reduce the high ink volume and opened the doors for the use of water-based inks by improving the ink drying rate and its adhesion on the substrate.

The reduction in the depth of engraving cell with improved cell geometry on LE cylinder minimized the transfer of high ink volume. The printing properties were checked to benchmark the performance of printing from EME and LE cylinders. The difference in consumption of raw materials used for copper and chrome-plating was intended from the study conducted at AGPL. This novel study highlighted the significance of copper and chrome-plating role and assessment of reduction of cost and CFP in gravure printing cylinder making process. Many studies were conducted for rotogravure printing and water-based ink, but none of the studies is reported for the reduction of carbon footprint and cost from raw materials and power consumption.

## Materials and methods

To bridge this extended technical gap, the present study was focused on the configuration of the cell geometry for electromechanical and laser engraved cylinders and compared the printing parameters and cost benefits. Two cylinders with having a circumference of 520 mm and a length of 1100 mm were taken for engraving by EMR and LE methods to compare the printing parameters. This study covered the reduction in consumption of plated materials and power along with cost-saving per cylinder. The CFP was calculated from the difference in the consumption of copper and chrome-plating processes and power consumption in rotogravure printing cylinder manufacturing units.

All the materials and chemicals used in this study were purchased by AGPL. Copper anode purchased from Luvata Corporation, Italy, chrome solution from Atotech Chemicals, Bengaluru, Sulphuric acid from Merck Chemicals, India, Ballard powder from MDC Max Daetwyler, Switzerland and other chemicals purchased from the local market. Inks used for printing the samples were purchased from Sakata inks, India. Corona treated Poly Ethylene Terephthalate (PET) film was purchased from Uflex Ltd, Noida.

### Sampling site and location

Delhi-NCR (National Capital Region of India) is the largest manufacturing base for flexible packaging laminates production facilities with high volume market demand, well-developed supporting vendors with basic amenities. AGPL is the largest cylinder-making unit of India with an export facility to many countries and all necessary trials and studies were conducted at AGPL. AGPL has well established and has a state-of-the-art facility for manufacturing rotogravure printing cylinders, flexographic plates and embossing rollers.

### Alternation of cell geometry and depth of engraving cell

**Figure 2:**
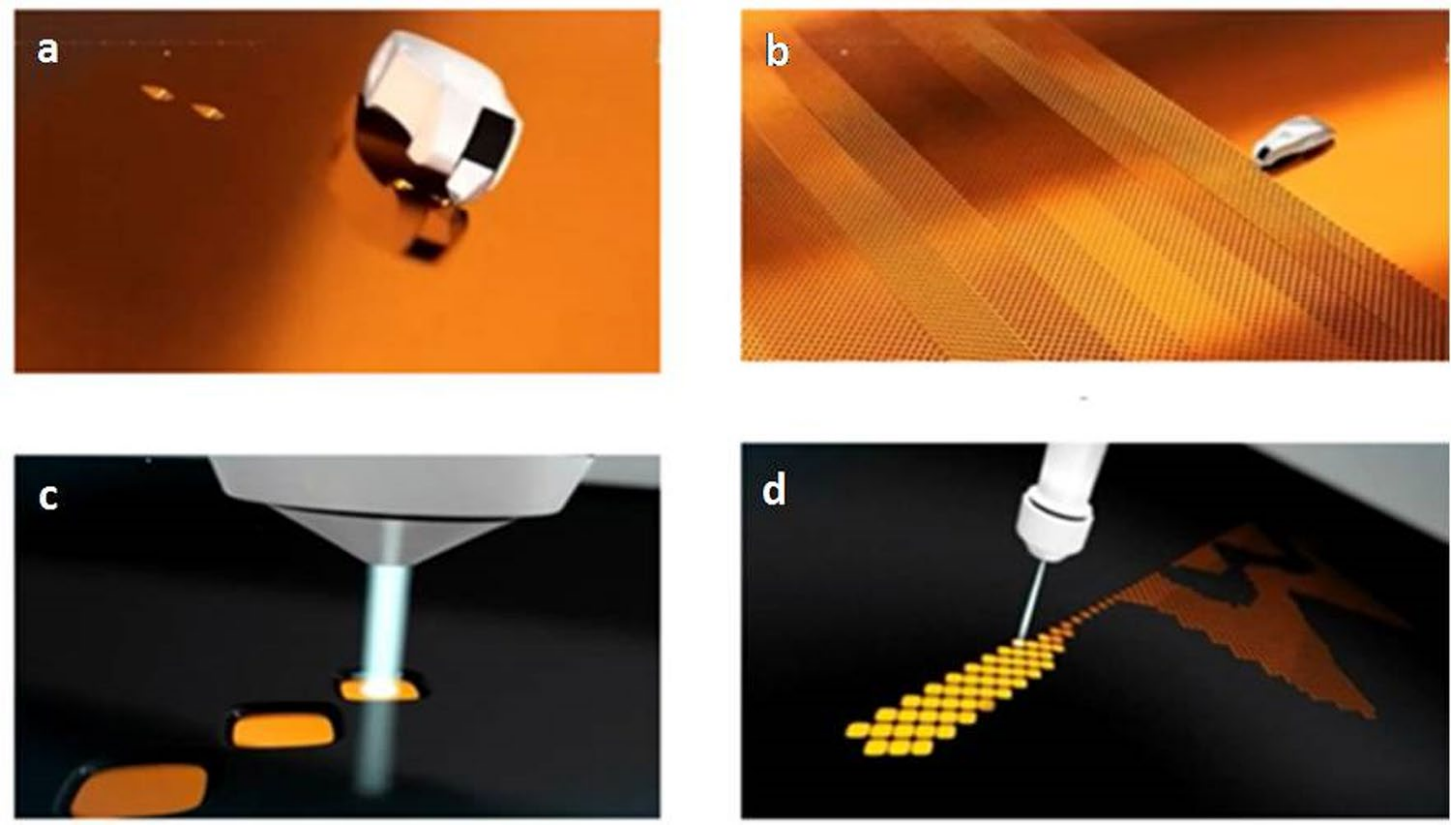
Images show (**a**) EME engraving process, (**b**) EME engraved cell on Cu plated cylinder, (**c**) laser engraving process and (**d**) laser engraved cells. Source: (youtube.com/watch?v=5hb3EKQv4ic).

In electromechanical engraving, control of cell depth was due to the mechanical process of the stylus which dig the cell on a finished copper surface^15^. Stylus needed some range of copper thickness so that it should not hit the mild steel surface of base cylinder due to ovality. Stylus, which dig the cell on cylinder is made of diamond and highly brittle, sharp, and costly. Any sudden impact may cause a break or loss of the sharpness of the diamond stylus. A stylus having a certain angle and length to dig the cell on the electroplated copper surface but damages in stylus are the prime source of cell depth variation^19^. But in laser engraving system, there is no mechanical or physical process of digging the cell on copper surface of the printing cylinder as shown in Fig. 2. Engraving or cell digging process is governed by laser beam. Electromechanical engraving has certain limitations because it could not provide a minimum (5 µm) and maximum (80 µm) cell depth as a laser can achieve.

Cell parameters played a significant role in achieving the transfer of ink volume on a substrate and this mechanism is governed by some parameters like cell depth, lines per inch, cell size, stylus, and copper-plating quality^20^. In this study, two cylinders were prepared, one cylinder was engraved by electromechanical engraving and the other by laser engraving method to study the minimum engraved cell depth, color values and shade within the standard range. Ballard skin method was used to study the thickness of copper and chrome-plating separately^21^.

### Cylinder preparation

Copper can not be directly plated on mild steel cylinders due to iron present in mild steel, so the surface of mild steel not having a bond with copper plating. So, cylinders were first fabricated on a lathe machine according to the dimensions of the pouch. The steel cylinders were electro-plated with nickel to get the final thickness ranging between 10 and 12 µm so that layer of Cu could be bonded to the mild steel base cylinder. The minimum thickness of Cu must be in the range of 150–200 µm as per the machine standard of MDC Max Daetwyler (Switzerland). A Cu layer of thickness of about 50 µm was polished to get a uniform surface with surface roughness (R_z_) ranging between 0.2 and 0.4 µm. After that, the Cu-plated polished surface was engraved with stylus in oscillating motion and hitting the Cu surface (Fig. 2a,b). A stylus is made of diamond and sharpened at the desired angle varying from 100° to 130° separately, which dig the Cu surface and engraved the cell.

### Preparation of ballard skin

In the Ballard skin test, 2 g of Ballard powder was mixed in one litre of deionised water and poured slowly on the entire surface of the cylinder before copper plating process on the semi-finished surface of copper. After completion of the chrome-plating process, the Ballard layer peeled off manually from the semi-finished surface of copper-plating. Ballard skin test was performed to check the copper and chrome-plating thickness for ECE and LE cylinders. Copper and chrome thickness was measured by FE-SEM (Mira 3Tescan) to evaluate the difference in thickness^21^.

### Comparison of printing properties

Printing was performed with Sakata ink by proofing machine (JM Heaford, UK) and printing parameters were studied to benchmark the printing performance from EME and LE cylinders printed ink.

Ink transferred on PET films formed as a film, thickness of ink layer by EME and LE cylinders were analysed by a Field Emission Scanning Electron microscopy (TESCAN MIRA3). Following parameters were measured for comparative study of both engraving systems.

### Thickness of printed ink

Transferred ink thickness on printing substrate on PET film by EME and LE cylinders was analysed by a Field Emission Scanning Electron microscopy (TESCAN MIRA3, USA) operating at an acceleration voltage of 10–20 kV. After ink transfer, printed samples were cut into the required size of the sample and kept in the sample holder. After that samples were sputter-coated with gold before the FE-SEM experiment started.

### Measurement of color strength

Ink specific color components strength (L*, a*, b*) of the image transferred on printing substrate (PET film) were determined by Spectrox Eye spectrophotometer. The ink printed on PET films was measured for color coordinates (LAB values) at randomly chosen five distinct points as per the method given by^22^.

### Viscosity

Ink consistency was determined by using PAINTLAB + Viscomixer (Rhopoint Instrument Ltd, U.K). The viscomixer's probe was dipped into the 50 ml ink, and the viscosity of ink was measured in the triplicates as per method given by^23^.

### Surface energy

The surface energy of ink was determined by using Tensiometer (KSV Instruments Ltd, Finland) with rectangular platinum (Pt) plate method at 25°C. 12 g of ink was taken from both the inks and dissolved in a 24 ml solution of deionised water and IPA (80:20) for water-based ink and 24 ml of ethyl acetate for solvent-based ink. Both the inks were kept for 10 min on a magnetic stirrer for making a homogenous solution^24,25^.

### Adhesion study of ink on PET film

The adhesion of both the inks on the printing film surface was conducted by using the 3 M standard tape (1.5 cm) method. A standard tape having a width of 1.5 cm was stuck and pasted on PET film’s surface and pulled off manually. The removal of printed ink from the surface of the printed sample was observed. The percentage of adhesion (%) was calculated using the following equation:

1 $$\mathrm{Adhesion }(\mathrm{\%}) = \frac{\mathrm{A}}{\mathrm{A}0}*100$$

A_0_ and A signify the number of grids covered by the standard tape and rest by the ink, respectively^26,27^.

### Calculation of power consumption

Cu deposition on cylinder is principally governed by Faraday’s laws of electrolysis. Cu of purity 98–99% was deposited on a nickel-plated mild steel base cylinder. Standard parameters for the electroplating process were maintained at 50% immersion, an efficiency of 98%, and current density at 25 A/dm^2^ for plating the cylinder. The electrolyte of the Cu plating machine was maintained as copper sulphate (220–240 g/l) and H_2_SO_4_ (60–65 g/l) and hardener were used to increase the hardness of deposited Cu^28^. The consumption of Cu for the required thickness was calculated by using Eq. (2):

2 $$\mathrm{Copper} \, \mathrm{ weight}=\frac{\mathrm{thickness }*\mathrm{ surface} \, \mathrm{area }*\mathrm{ density}}{100}$$

where thickness represents the amount of deposited copper, the surface area is the area of cylinder and density represents the density of pure Cu.

Similarly, chromium deposition on ECE and LE printing cylinders was calculated conferring Faraday’s laws of electrolysis. The standard parameters for electroplating of chrome-plating were maintained at 50–55°C, current density—50 A/dm^2^, efficiency—21% and immersion 50%. Chrome-plating electrolyte was maintained as hexavalent chrome—250–280 g/l, trivalent chrome—5–10 g/l and sulphate content—2.5–5.0 g/l. The weight of chromium was calculated according to Eq. (1), where copper was replaced with chromium.

Power consumption was calculated during the process of electroplating of trial cylinders. General or ideal electric power consumption was not considered but power consumed by the rectifier for the electroplating process was studied. The total current in amperes required for plating of one cylinder was calculated by using the following Eq. (3):

3 $${\text{Current} \, \text{in} \, \text{Amp}} = {\text{ circumference }}*{\text{length }}*{\text{ immersion}}*{\text{current} \, \text{density}}$$

Here, immersion was the % area of cylinder dipped in the electrolyte, current density for copper 25 A/dm^2^and for chromium 50 A/dm^2 ^as per ISO standard.

The power consumption in Ah is calculated according to the following equation;

4 $$\mathrm{Power} \, \mathrm{consumption} \, \mathrm{in} \, \mathrm{Ah }= \frac{\mathrm{Weight }*100}{\mathrm{ECE }*\mathrm{ Efficiency}}$$

Weight was the consumed quantity of anode, ECE is electrochemical equivalence and efficiency is the % of machine set at the scale of 100%.

Plating time for one cylinder can be calculated by using the following equation.

5 $${\text{Plating}}\;{\text{time}}\;{\text{for}}\;{\text{one}}\;{\text{cylinder}} = \frac{{{\text{Time }}\;{\text{in}}\;{\text{second}}*{\text{Ah}}}}{{{\text{Current}}\;{\text{in}}\;A}}$$

### Reduction in carbon footprints

AGPL manufactured approximately 1,65,000 cylinders annually and out of which 40% were produced by laser engraving process. In the present study, 60,000 cylinders were taken for total carbon footprints and calculated for the reduction in plated materials quantity and power consumption during manufacturing. Another company X is also having almost same production capacity in Noida plant for EME and LE cylinder manufacturing, the outcome of this study was also calculated.

CFP was calculated by using following equation.

6 $${\text{P }} = {\text{ I }} \times {\text{ V}}/{1}000$$

where, P was in kW, I was the current (I) in amps (A), and V was the voltage in volts (V) (https://www.rapidtables.com).

The CFP for copper was calculated according to the factor given by Chesnokov et al. and the factor for CFP of chromium was taken from the standard factors given for CO_2_ emissions^13^. Carbon footprints were calculated as a reduction in power consumption according to Eq. (7):

7 $${\text{Output }}\;\;{\text{value}}\;{\text{ in }}\;\left( {{\text{kg of CO}}_{{2}} } \right) \, = {\text{ Input }}\;{\text{value }}\left( {{\text{in }}\;{\text{kWh}}/{\text{year}}} \right) \, * \, 0.{85 }\left( {{\text{Emission}} \;{\text{factor}}} \right)$$

### Consent to participate

All authors consent to participate.

### Consent for publication

All authors consent to publish.

## Results and discussion

### Difference in consistency of engraving cell

In rotogravure printing, the geometry of engraved cells played a key role in transferring the ink on printing substrate. In EME, the cell was helical in shape which could not be changed while in laser engraving, it was possible to modify the shape of the engraving cell. In an engraving process, lines per inch (LPI) and dots per inch (DPI) played an imperative role in the quality and clarity of a printed image. LPI referred to the lines in a one-inch area of a halftone or screen. Higher the LPI, the smaller the screen size due to covering the area by dots. DPI was the resolution of a print image indicating the number of dots per inch printed on a substrate. The more the dots, the finer will be the printing.

The depth of the laser engraved cells was reduced due to a reduction in the depth of Cu and Cr plating compared to electromechanical engraved cells. Therefore, laser cells contained less quantity of ink during printing and transferred the same quantity of ink on the substrate. To achieve this quality, all the cells engraved on the surface of copper, must transfer the ink on the printing substrate. When few of the cells fail to transfer the ink on printing substrate during printing process is known as dot missing.

**Figure 3:**
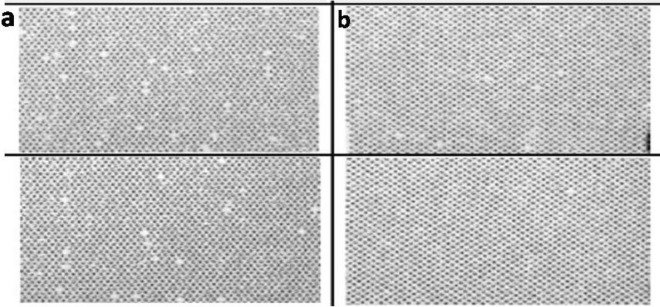
(**a**) Missing dots in EME printing (**b**) Less missing dot in LE printing.

Figure 3 shows that cell missing was also observed less in LE engraving, which was good for text and halftone printing due to an increase in dot transfer.

### Surface morphology and thickness of copper and chromium plating

**Figure 4:**
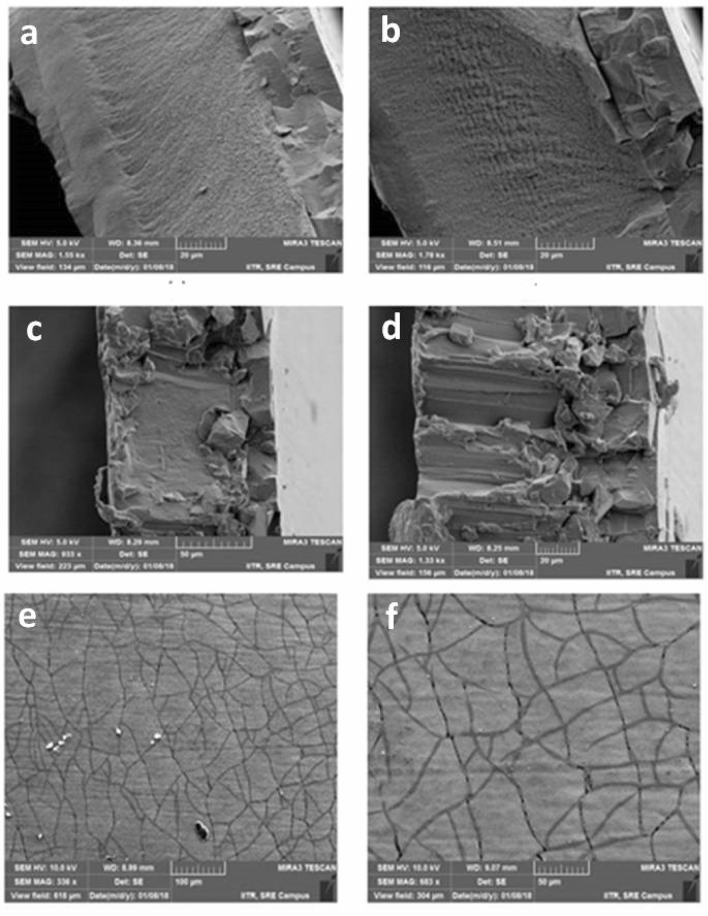
FE-SEM images of Ballard skin test Cu and chrome-plated cylinder’s surface (**a**, **b**) shows the left and right sides of LE cylinder’s copper and chrome thickness, (**c**, **d**) shows the left and right sides of EME cylinder’s copper and chrome thickness, and (**e**, **f**) shows the cracks on chrome surface.

Ballard skin test was conducted to measure the surface properties and thickness of Cu and chrome plated EME and LE cylinders. The thickness in EME cylinder plated with Cu ranged from 145 to 195 µm whereas the thickness of the chrome-plated cylinder was measured between 9.5 and 11.5 (Fig. 4a,b).The thickness of Cu in the LE cylinder varied between 75 and 110 µm and 4.5–6.5 µm for chrome-plated cylinder (Fig. 4c,d). The surface of chrome-plated cylinder had a good number of cracks, which helped the cylinder for a long printing life. Chrome cracks generally provided lubrication through tiny canal of chrome cracks (Fig. 4e,f) to doctor blade while squeezing the ink from the chrome surface during printing.

### Comparison of printing quality

Printed samples from EME and LE engraved cylinders were compared for the post-printing properties and required quality parameters for printing were examined for flexible packaging as per existing industry parameters.

### Thickness of printed ink

**Figure 5:**
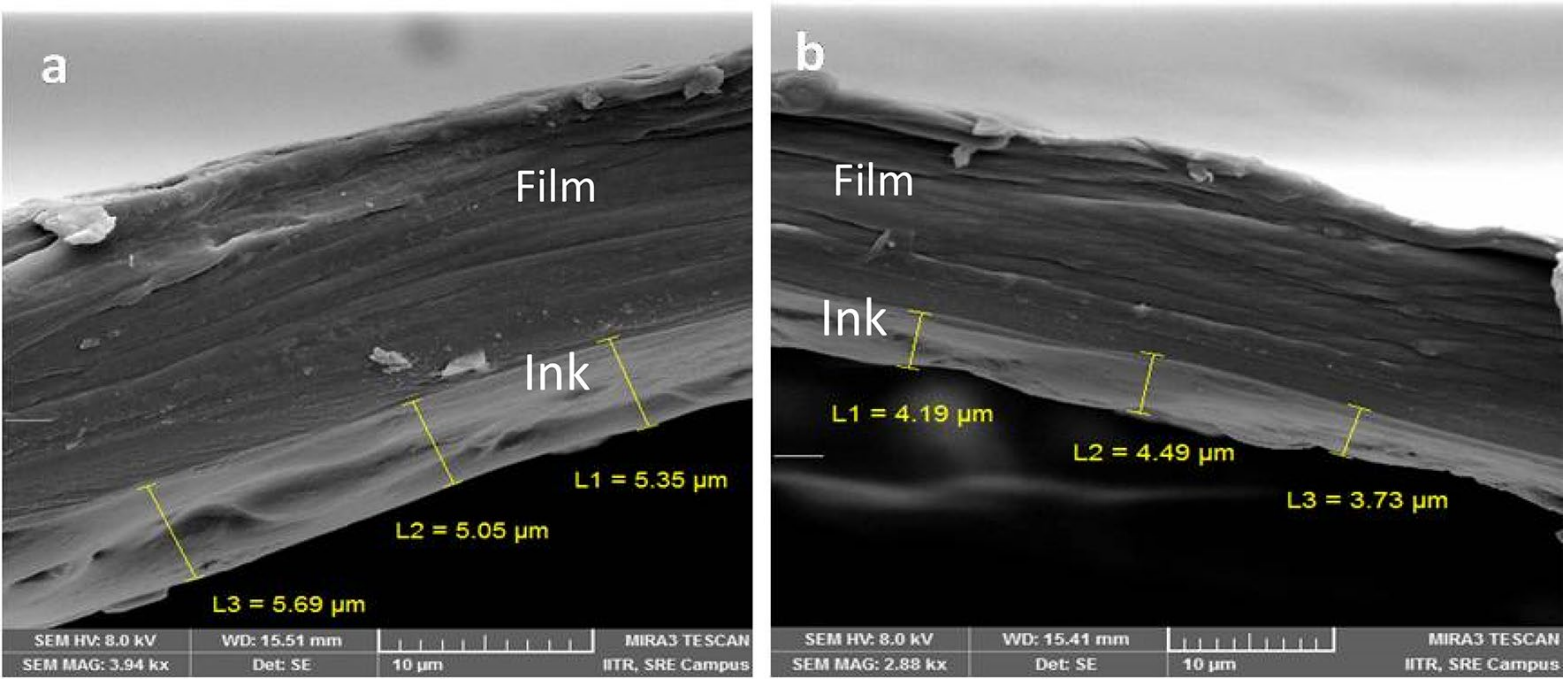
(**a**) Shows the printed ink deposition by EME, and (**b**) shows the ink deposition by LE cylinder.

Printing inks thickness transferred in printing process on polymer film reflected as a very substantial property to achieve the targeted color values. Gloss, brightness, pigment distribution and LAB values were depended on the thickness of transferred ink. The thickness of printed ink on substrate should be maintained between 3 and 6 µm for printing applications. Pigments dispersed upon the surface gave the appropriate color values. Further, the thickness of the printed ink layer on the PET film was also measured by FE-SEM spectroscopy (TISCON) (Fig. 5) which showed that the developed inks demonstrated good film-forming properties on the PET film with the essential thickness of ink. Average ink thickness imparted by EME and LE cylinders was measured 5.36 µm and 4.13 µm, respectively.

### Viscosity

Viscosity plays a vital in the chemistry of water-based ink due to ink transfer from the engraving cell is strongly depended on flow behaviour of ink. The viscosity of the inks used for the printing trial was measured 40 ± 1.3 cp, 40 ± 1.6 cp, and 25 ± 1.28 cp for black, cyan and yellow inks, respectively. Viscosity of printing inks played a dynamic role during printing operation because ink composition and solid content influenced ink viscosity. Frequent changes in the viscosity of ink might affect the desired color and shade during printing process adversely. Viscosity was concerned with a fluid’s resistance to flow, changing with temperature and agitation or rate of flow and it directly influenced printing quality, printability, drying time, adhesion, gloss, etc. Most of the printing units had an online monitoring facility to control viscosity by the addition of solvent^2^.

### Surface energy

The surface tension of inks must be kept lower compared to the wetting tension of substrate for getting good printability, adhesive bonding, or distribution of ink. Surface tension of printing ink ensured the wetting of substrate, spreading over and retracting from the substrate. Ink had both adhesive and cohesive forces which determined the extent of association and self-adhesion to the substrate respectively. Typically, the surface tension of solvent inks was in the 20–30 dynes/cm range^29^. Results show that surface tension of printing inks was measured in between the range of 26–28 dyne/cm. These values were related to the good adhesion properties of ink on PET films.

### Adhesion

**Figure 6:**
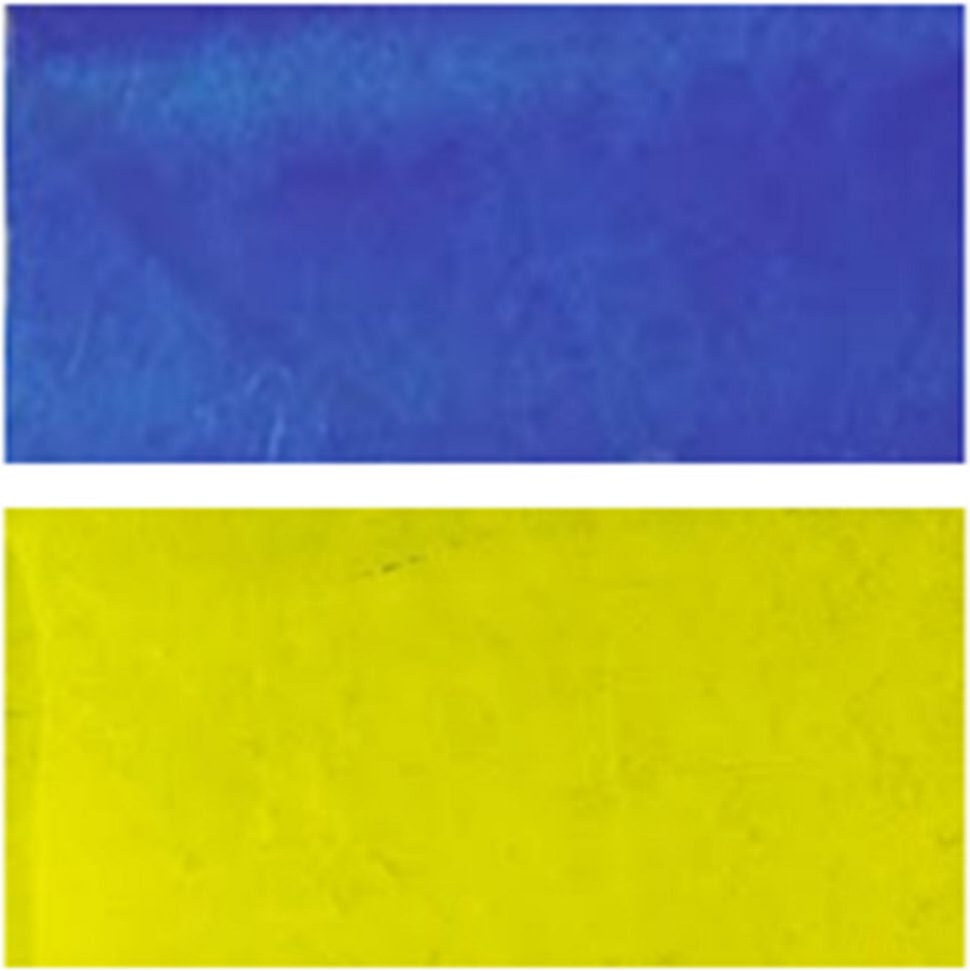
Shows the adhesion of printed ink on PEL film.

Bonding of ink composition with plastic film is a very critical property of ink due to readability and visibility of printed material are subject to legal concern. Adhesion of ink on the printing substrate decided the adhesion period of printed ink. In surface printing, adhesion is a very critical parameter because it interacted with the other impacting surfaces directly. As shown in Fig. 6, 70–80 ± 4% adhesion was measured from trial ink for yellow and cyan ink by standard 3 M tape (width, 1.5 cm). Rub resistance and scuff resistance are highly depended on the physical and chemical bonding between the ink and printing substrate. These results showed that by reducing the cell depth, adhesion of ink on printing substrate did not reduce in comparison to EME printing samples^30^.

### Color values of inks

**Figure 7:**
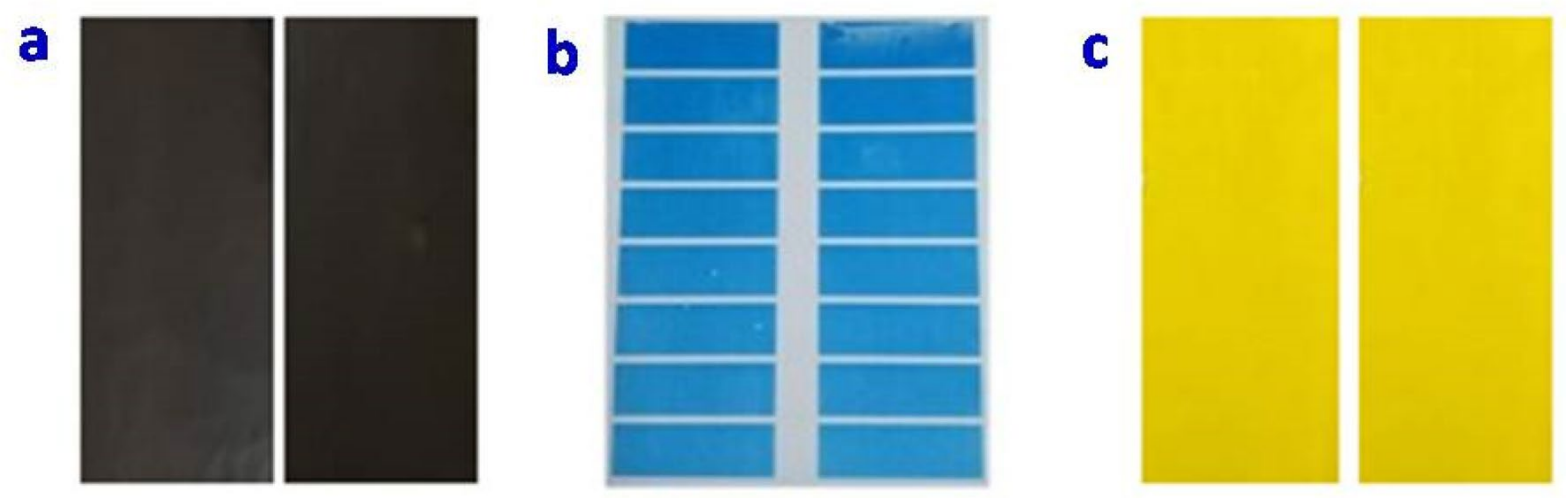
(**a**) Black, (**b**) cyan, and, (**c**) yellow inks sample respectively printed on LE and EME cylinders.

Color is strongly influenced by the pigment concentration and the distribution of pigment particles. Color is the inherent property of any ink, which was directly related to aesthetics and delivered all the legal compliance information to the consumer. The L*, a*, and b* values of printed PET films are shown in Table 1 and Fig. 7. L* value of black ink showed lightness, and no change in cyan ink but yellow inks showed a marginal increase*, on LE printed samples compared to EME printed samples. a*, and b* showed light changes in color zone of LE printed samples compared to EME printed samples. The high L* values for all the inks indicated good color strength and print quality on the corona-treated PET films^31^. Results show that ink transferred by both the engraving system is complying with the required LAB values.

**Table 1:** LAB values of black, cyan and yellow inks.

Sl. no.	Parameters	EME printed sample			LE printed samples		
		L*	a*	b*	L*	a*	b*
1	Black	15 ± 0.24	1 ± 0.02	3 ± 0.47	13 ± 0.48	0 ± 0.02	2 ± 0.011
2	Cyan	19 ± 0.37	9 ± 0.36	− 30 ± 0.68	19 ± 0.22	14 ± 0.18	− 39 ± 0.72
3	Yellow	82 ± 1.23	7 ± 0.38	82 ± 1.15	84 ± 1.16	7 ± 0.27	84 ± 1.12

### Influence on printing properties

**Figure 8:**
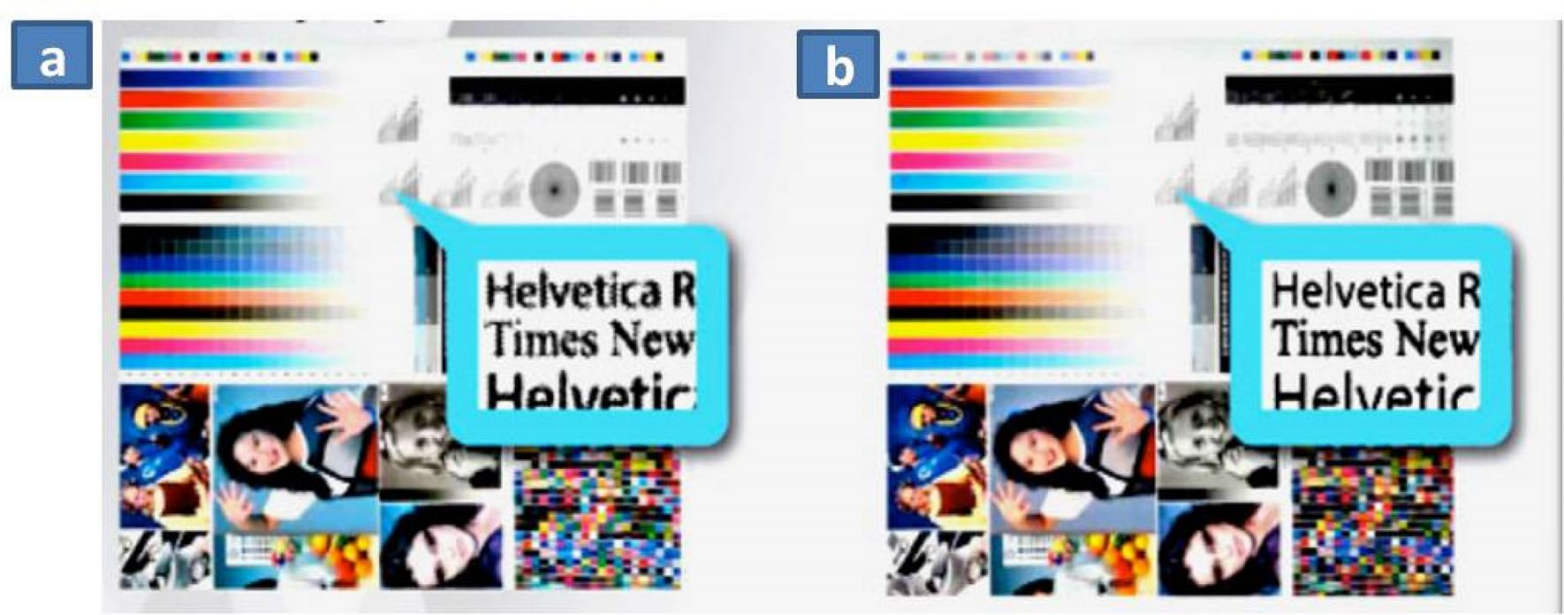
Proofing results from (**a**) EME method (**b**) LE method.

Figure 8 shows that the readability and sharpness of text matter were better in LE printed samples compared to EME process. It was also observed that text readability was better in laser engraved cylinders due to even cell and consistent dot transfer (Fig. 8), whereas in EME cylinder text is not as clear. Difference in this arises due to wear and tear condition of engraving stylus, which digs the non-uniform cell.

### Reduction in consumption of copper and chromium in LE and EME cylinder

Metals are precious and Cu and Cr are used in the cylinder making process. Due to present global issues, prices of all the metals are in increasing trend. So, industries are forced to reduce the operating cost without compromising the quality of the product. Data were compared for the manufacturing process and raw materials required for both the process. The LE cylinder of size 520 mm × 1100 mm showed a reduction in Cu by 43.83% compared to EME cylinder. The plating on LE cylinder required 38.44 g less copper anode per cylinder compared to EME cylinder (Table 2). Cr-plating was required to protect the engraved surface of Cu and stand under the pressure of doctor blade during printing. The thickness for Cr-plating was maintained between 10 and 12 µm for EME cylinder as per the ISO standard, whereas the thickness for Cr-plating was between 4 and 6 µm in case of LE cylinder. A high Cr thickness was required for EME cylinder due to higher cell depth. The consumption of Cr was 45.3 g for EME cylinder and it was reduced to 20.6 g for LE cylinder. Difference in consumption of copper and chrome metal was not compromising the quality of printing.

**Table 2:** Consumption of copper and chrome during plating.

Engraving types	Circumference (mm)	Length (mm)	Total thickness, (µm)	Density (g/cm^3^)	Copper consumption (kg)
**Copper-plating**					
EME	520	1100	170 ± 5	8.96	0.87 ± 0.03
LE	520	1100	95 ± 2	8.96	0.49 ± 0.02
Copper saved per cylinder					0.38 ± .03
**Chrome-plating**					
EME	520	1100	11 ± 0.5	7.19	0.045
LE	520	1100	5 ± 0.2	7.19	0.020
Chrome saved per cylinder					0.024

The Cu-plating of LE cylinder showed a reduction in power consumption by 327.40 Amp-h and time 29.3 min compared to plating of EME cylinder. While Cr-plating showed a reduction in power consumption by 363.96 Amp-h and time 15.3 min (Table 3). The time saving might be used to improve productivity without increasing any manpower, and investment to improve infrastructure. Zhang showed that reduction in thickness during plating of EME and LE cylinders also reduced plating material and time^32^. This study concluded that use of the LE method is very beneficial for raw materials and power consumption, thus adopting this recent technological development for manufacturing of rotogravure printing cylinder method will be favouring industries.

**Table 3:** Saving in power consumption and time for copper and Cr-plating of EME and LE cylinders.

Engraving type	Circumference (mm)	Length (mm)	Plating thickness (µm)	Current (A)	Ah	Time (min)
**Copper-plating**						
EME	520	1100	170 ± 5	715 ± 6.17	742.40 ± 0.35	64.1 ± 0.50
LE	520	1100	95 ± 2	715 ± 5.86	415 ± 0.28	34.8 ± 0.35
Saving in power consumption and time					327.40 ± 0.29	29.3 ± 0.43
**Chrome-plating**						
EME	520	1100	11 ± 0.5	1430 ± 8.46	667.26 ± 0.38	28 ± 0.26
LE	520	1100	5 ± 0.2	1430 ± 9.23	303.30 ± 0.42	12.7 ± 0.15
Saving in power consumption and time					363.96 ± 0.39	15.3 ± 0.19

### Cost saving in LE cylinder

Globally industries are facing lots of challenges like moving towards automization, 4.0, increase in raw materials cost as well employee cost. So, it is the need of the hour for industries to reduce the operating cost and move for cost saving projects. Reduction in cost for producing high-quality products is the key to continual growth for any organization as well as its employees also. It is also noteworthy that when the thickness of copper plating reduces then the demand for power and other chemicals also reduces. Table 4 shows that the Cu and Cr-plated LE cylinder saved US$ 4.08 per cylinder compared to EME cylinder. If AGPL manufactured 60000 LE cylinders per annum, the total saving will be US$ 27,502.2 per annum and power saving will be US$ 376.2 compared to EME cylinder^33^.

**Table 4:** Total cost saving per cylinder from raw materials and power consumption saved.

Parameters	Plated materials saved in kg	Cost in US$	Power consumption, kA*	Cost in US$*
**Copper-plating**				
EME-cylinder	0.87 ± 0.03	8.35 ± 0.30	0.74	0.074
LE-cylinder	0.48 ± 0.02	4.70 ± 0.29	0.42	0.042
Saving	0.38 ± 0.02	6.65 ± 0.29	**0.32**	**0.032**
Chrome-plating*				
EME-cylinder	0.045	0.67	0.67	0.067
LE-cylinder	0.020	0.30	0.42	0.042
Saving	0.024	0.37	0.25	0.025
Total cost of Cu and Cr-plated EME cylinder				9.16
Total cost of Cu and Cr-plated LE cylinder				5.08
**Total saving in Cu and Cr-plated LE cylinder**				**4.08**

Rate of Cu = US$ 9.60 per kg, rate of Cr = US$ 15.08 per kg and rate of electricity US$ 0.10 per unit. (Conversion rate: One US$ = ₹72.99 as of 03.09.2021).*SD for power consumption, chrome plating and cost was measured in 3 decimal points.

**Figure 9:**
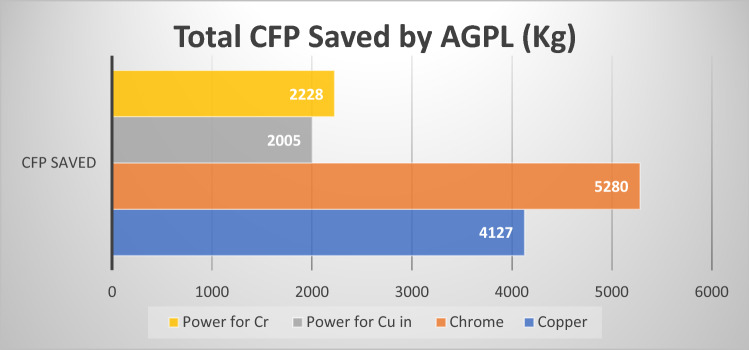
Total CFPs saved during laser engraving process.

Figure 9 shows that a total 13,639 kg of carbon dioxide was reduced by AGPL after 40% conversion EME cylinders to LE cylinders (production of 60,000 cylinders from LE). The maximum CFP were saved in chrome electroplating process and minimum in CFPs in copper-electroplating. These results demonstrated that industries can reduce the operating cost for manufacturing high end product, which not only reducing the cost but also reducing the carbon footprint.

## Uncertainty analysis

In this present research work, there were some uncertain factors analysed which were due to the lack of related parameters and various limitations. We monitored and analysed different sizes of cylinders for conducting the trial. Cylinder circumference varies from 400 to 1100 mm and length has a variation from 800 to 1400 mm in routine production.

During the study period, electrolyte used for plating purposes was maintained as per the testing standards of ISO. SOPs were followed to prepare the trial cylinder and conducting the trial at several steps as per Fig. 1. One cylinder was prepared in one day in all three working shifts to improve the monitoring results, accuracy and precision of parameters maintained at the site. Printing from prepared cylinder (Proofing) was taken at room temp and 50% relative humidity to get the consistent ink transfer on PET films. All calibration certificates of monitoring and measuring devices and masterpiece of calibration tool were evidenced by authorized certification agencies. These measures were taken to eliminate the impact of circumstantial issues on monitored results to a certain level to reduce the uncertainty factor of this trial work.

## Conclusions

This study reveals that by adopting the LE process, the thickness of Cu and Cr layers could be reduced to 75 µm and 5 µm respectively. Raw materials and power consumption was also compared and data shows that LE process is having cost saving advantages over LE process. By converting 40% of production by laser engraving, data of AGPL confirmed that 13,639 kg emission of CO_2_ could be reduced. It means producing one cylinder will save 227 g of CO_2_ emissions in the environment. In this way, it contributed to green and sustainable production activity and favoured a better environment for humans.

Based on derived results and conclusions in the manufacturing of rotogravure printing cylinders, cylinder-making companies could contribute to environment-friendly, competitive, and sustainable production in the future. This advanced technology for LE cylinder required to aware by other business owners, managers and employers of printing and packaging units. The outcome of the presented model can facilitate government authorities to take futuristic decisions to reduce the CFP and cost from other units of cylinder making units in India.

## Abbreviations

EME: Electro mechanical engraving
Cu: Copper
DPI: Dots per inch
ECE: Electro chemical equivalent
AGPL: Afflatus gravures private limited
LPI: Lines per inch
IPA: Iso propyl alcohol
LE: Laser engraving
Cr: Chromium or chrome
WB: Water-based
CFP: Carbon footprint
ISO: International organization for standardization
SB: Solvent-based
VOC: Volatile organic compounds
